# On the Nervous Systems of Animal and of Vegetative Life, Their Anatomical Connexions, and Their Physiological, Psychological, and Zoological Relations

**Published:** 1841-10

**Authors:** 


Art. XIII.—*Du Système Nerveux de la Vie Animale, et de la Vie Vegetative; de leur Connexions Anatomiques, et des Rapports Physiologiques, Psychologiques, et Zoologiques, qui existent entre eux.* Par A. Bazin, d.m.p., Professeur de Physiologie Animale, et de Zoologie, à la Faculté des Sciences de Bordeaux, &c. &c.

*On the Nervous Systems of Animal and of Vegetative Life, their Anatomical Connexions, and their Physiological, Psychological, and Zoological Relations.* By A. Bazin, d.m.p., Professor of Animal Physiology and of Zoology to the Faculty of Sciences at Bordeaux, &c. —*Paris*, 1841. 4to, pp. 180. *With Five Plates.*

The title of this work, and the promises set forth in the preface, joined to the reputation which its author has attained, caused us to anticipate much interest in the perusal of it. This anticipation, however, has not been by any means fully realized. Many doctrines are put forth as novel which have long been familiar to physiologists under a form but little different, and that which there is of novelty rather consists of vague speculation than of real scientific induction. The work contains, however, much that is in itself sound and valuable; but this is frequently expressed in a form so diffuse, as to prevent its merit from being at once discovered. This is the case, more especially with the portion of the work in which the actions respectively due to instinct and to intelligence are under discussion.

The anatomist will find in this work a very full account of the structural connexions which exist between the cerebro-spinal and sympathetic systems in man; and there is a statement which, if true, is certainly important, that the pituitary gland is really a ganglion belonging to the sympathetic system. But we do not perceive that any such general deductions are drawn from these facts, as we were led to expect by the author's introductory remarks. Some other interesting observations are scattered through the work; such as, that there is a decussation between the fibres that connect the cerebrum and cerebellum, which we do not recollect to have seen noticed. And on the whole, we regard the anatomical portion as more deserving of attention than the physiological, which is in many parts very deficient; we have looked in vain, for instance, for any clear enunciation of the author's views of the influence of the ganglionic system on "la vie vegetative," although the subject is frequently alluded to.

